# Living Well with Kidney Disease by patient and care-partner empowerment: Kidney Health for Everyone Everywhere

**DOI:** 10.1007/s40620-021-01000-6

**Published:** 2021-03-06

**Authors:** Kamyar Kalantar-Zadeh, Philip Kam-Tao Li, Ekamol Tantisattamo, Latha Kumaraswami, Vassilios Liakopoulos, Siu-Fai Lui, Ifeoma Ulasi, Sharon Andreoli, Alessandro Balducci, Sophie Dupuis, Tess Harris, Anne Hradsky, Richard Knight, Sajay Kumar, Maggie Ng, Alice Poidevin, Gamal Saadi, Allison Tong, for the World Kidney Day Steering Committee

**Affiliations:** 1 The International Federation of Kidney Foundation – World Kidney Alliance (IFKF-WKA), Division of Nephrology, Hypertension and Kidney Transplantation, University of California Irvine, Orange, CA USA; 2 Department of Medicine and Therapeutics, Carol and Richard Yu PD Research Centre, Prince of Wales Hospital, Chinese University of Hong Kong, 30–32 Ngan Shing Street, Shatin, New Territories, Hong Kong, China; 3 Division of Nephrology, Hypertension and Kidney Transplantation, Department of Medicine, University of California Irvine School of Medicine, Orange, CA USA; 4 Tanker Foundation, Chennai, India; 5 Division of Nephrology and Hypertension, 1St Department of Internal Medicine, AHEPA Hospital, Aristotle University of Thessaloniki, Thessaloniki, Greece; 6 Hong Kong Kidney Foundation and the International Federation of Kidney Foundations – World Kidney Alliance, The Jockey Club School of Public Health and Primary Care, The Chinese University of Hong Kong, Hong Kong, China; 7 Renal Unit, Department of Medicine, College of Medicine, University of Nigeria, Ituku-Ozalla, Enugu, Nigeria; 8 James Whitcomb Riley Hospital for Children, Indiana University School of Medicine, Indianapolis, IN USA; 9 Italian Kidney Foundation, Rome, Italy; 10 World Kidney Day Office, Brussels, Belgium; 11 Polycystic Kidney Disease Charity, London, UK; 12 American Association of Kidney Patients, Tampa, FL USA; 13 Hong Kong Kideny Foundation, Hong Kong, China; 14 Department of Internal Medicine, Faculty of Medicine, Nephrology Unit, Cairo University, Giza, Egypt; 15 Sydney School of Public Health, The University of Sydney, Sydney, NSW Australia

**Keywords:** Patient empowerment, Care-partner, Low-middle-income countries, Health policy

## Abstract

Living with chronic kidney disease (CKD) is associated with hardships for patients and their care-partners. Empowering patients and their care-partners, including family members or friends involved in their care, may help minimize the burden and consequences of CKD-related symptoms to enable life participation. There is a need to broaden the focus on living well with kidney disease and re-engagement in life, including an emphasis on patients being in control. The World Kidney Day (WKD) Joint Steering Committee has declared 2021 the year of “Living Well with Kidney Disease” in an effort to increase education and awareness on the important goal of patient empowerment and life participation. This calls for the development and implementation of validated patient-reported outcome measures to assess and address areas of life participation in routine care. It could be supported by regulatory agencies as a metric for quality care or to support labeling claims for medicines and devices. Funding agencies could establish targeted calls for research that address the priorities of patients. Patients with kidney disease and their care-partners should feel supported to live well through concerted efforts by kidney care communities including during pandemics. In the overall wellness program for kidney disease patients, the need for prevention should be reiterated. Early detection with a prolonged course of wellness despite kidney disease, after effective secondary and tertiary prevention programs, should be promoted. WKD 2021 continues to call for increased awareness of the importance of preventive measures throughout populations, professionals, and policy makers, applicable to both developed and developing countries.

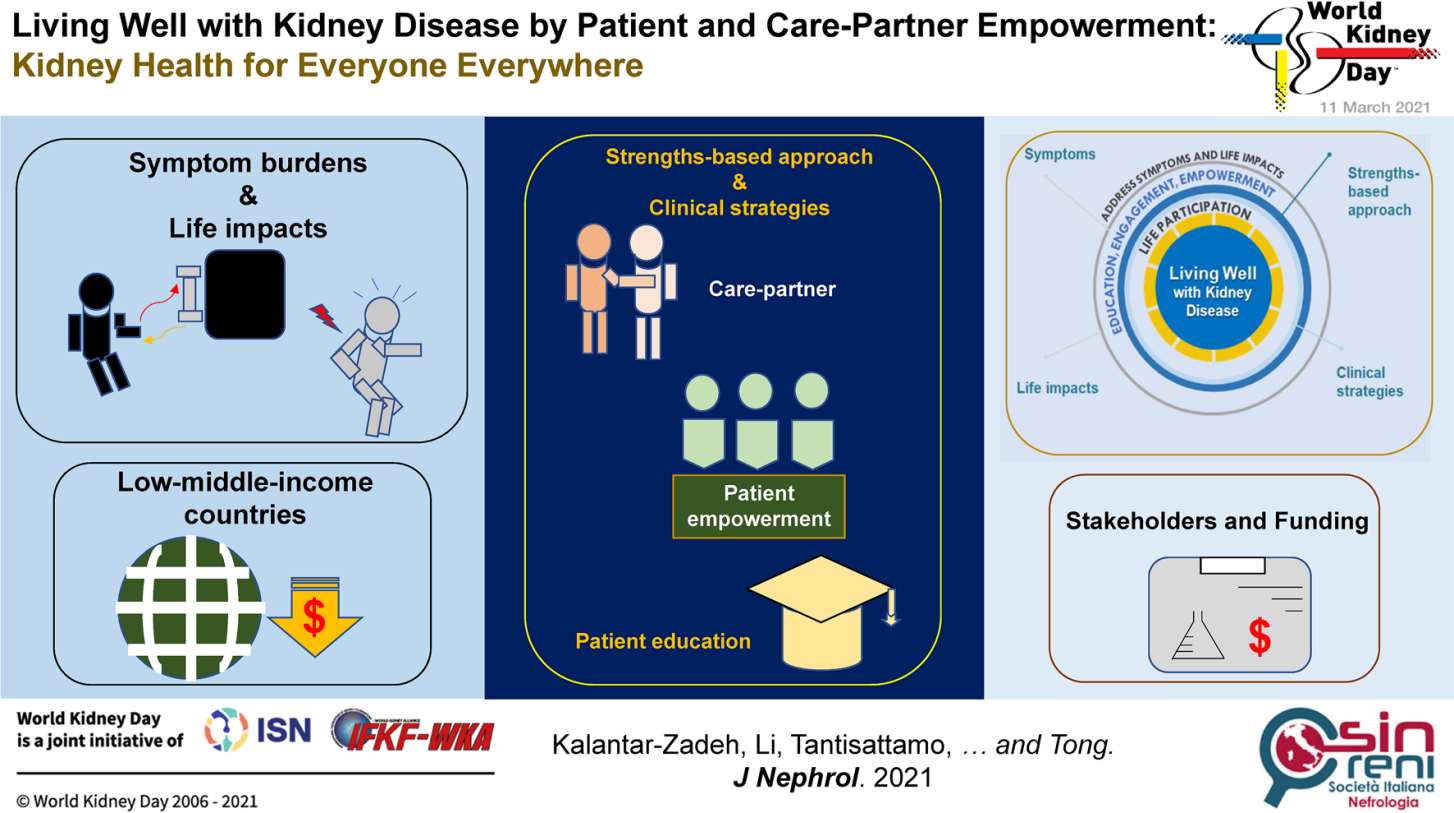

## Patient priorities for living well: a focus on life participation

CKD, its associated symptoms, and its treatment, including medications, dietary and fluid restrictions, and kidney replacement therapy can disrupt and constrain daily living, and impair the overall quality of life of patients and their family members. Consequently, this can also impact treatment satisfaction and clinical outcomes [1]. Despite this, the past several decades have seen limited improvement in the quality of life of people with CKD [1]. To advance research, practice, and policy, there is increasing recognition of the need to identify and address patient priorities, values, and goals [1].

**Fig. 1:**
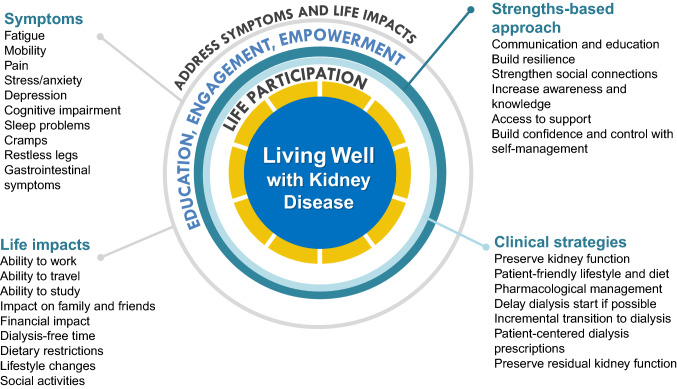
Conceptual framework of “Living Well with Kidney Disease” based on patient centeredness and empowering the patient with focus on effective symptom management and life participation

Several regional and global kidney health projects have addressed these important questions including the *Standardised Outcomes in Nephrology* (SONG) with more than 9000 patients, family members, and health professionals from over 70 countries [2, 3]. Across all treatment stages, including CKD, dialysis and transplantation, SONG participating children and adults with CKD consistently gave higher priority to symptoms and life impacts than health professionals did [2, 3]. In comparison, health professionals gave higher priority to mortality and hospitalization than did patients and family members. The patient-prioritized outcomes are shown in Fig. 1. Irrespective of the type of kidney disease or treatment stage, patients wanted to be able to live well, maintain their role and social functioning, protect some semblance of normality, and have a sense of control over their health and wellbeing.

*Life participation*, defined as the ability to do meaningful activities of life including, but not limited to, work, study, family responsibilities, travel, sport, social, and recreational activities, was established as a critically important outcome across all treatment stages of CKD [1, 2]. The quotations from patients with kidney disease provided in Box 1 demonstrate how life participation reflects the ability to live well with CKD [4]. According to the World Health Organization (WHO), participation refers to “involvement in a life situation” [5]. This concept is more specific than the broader construct of quality of life. Life participation places the life priorities and values of those affected by CKD and their family at the center of decision making. The World Kidney Day Steering Committee calls for the inclusion of life participation, a key focus in the care of patients with CKD, to achieve the ultimate goal of living well with kidney disease. This calls for the development and implementation of validated patient-reported outcome measures that could be used to assess and address areas of life participation in routine care. Monitoring of life participation could be supported by regulatory agencies as a metric for quality care or to support labeling claims for medicines and devices. Funding agencies could establish targeted calls for research that address the priorities of patients, including life participation.

**Box 1:** Quotations from patients with CKD related to priorities for living well

“I don’t want to think about dying from my disease. I want to be able to live well with my disease.”—Patient with CKD
“Life participation is most important because without it, you can’t do anything.” —Child with CKD
“Maybe it’s as simple as asking patients whether, how well they are able to participate in the life that they want to lead because it’s going to be different for different people”—Kidney transplant recipient
“Everyone has to face death, what I would like to have is a good quality of life rather than to face death.”—Kidney transplant recipient
“So, it doesn't actually really matter what the numbers say, and some of my numbers should have suggested that I should be feeling a lot worse than what I actually was, it's about how much I feel I can do and participate in my life and feel normal.” Patient with CKD
“I’m still living. I get out of bed, and I’m still living and still breathing. As long as I can do that, I’m going to carry on and be positive because life is short.”—Patient with CKD
“I put life participation because I know that looking from the outside, I know [his kidney disease] stops [him] from thinking bigger...Although that’s really big, there’s this life that has to happen at the same time.”—Family member
“Amazed at comments from professional(sic) about travel, free time, etc. they seem to think the mechanics of dialysis far more important. Dialysis is a treatment which keeps us alive to live a life, not just to wait for death.—Patient receiving dialysis
“I prefer to be above ground, then below ground. So why not enjoy life whilst being above ground.” Adam Martin
“Over the years, I have learned to worry less, control my emotions, and not fear death. I keep my mind active. I follow the advice of the philosopher-emperor Marcus Aurelius to 'love the hand that fate (has dealt me) and play it as (my) own'. Living well with CKD means to live the best life I can in the time I have available….Living well with CKD is the same as living well.”—Tess Harris
“While CKD brings me some limitations, I can maximize the possibility to live well. I kept working when I was doing hemodialysis. After transplant, I could live: study, work, travel, marry, have children, and service the community.”—Maggie Ng

*Personal communication; quotations are identified by name with permission

## Patient empowerment, partnership and a paradigm shift towards a strengths-based approach to care

Patients with CKD and their family members including care-partners should be empowered to achieve the health outcomes and life goals that are meaningful and important to them. The WHO defines patient empowerment as “a process through which people gain greater control over decisions or actions affecting their health,” [6] which requires patients to understand their role, to have knowledge to be able to engage with clinicians in shared decision-making, skills, and support for self-management. For patients receiving dialysis, understanding the rationale for a lifestyle change, having access to practical assistance and family support promoted patient empowerment, while feeling limited in life participation undermined their sense of empowerment [7].

The World Kidney Day Steering Committee advocates for strengthened partnership with patients in the development, implementation, and evaluation of interventions for practice and policy settings, that enable patients to live well with kidney diseases. This needs to be supported by consistent, accessible, and meaningful communication. Meaningful involvement of patients and family members across the entire research process, from priority setting and planning the study through to dissemination and implementation, is now widely advocated [8]. There have also been efforts, such as the *Kidney Health Initiative*, to involve patients in the development of drugs and devices to foster innovation [9].

We urge for greater emphasis on a strengths-based approach as outlined in Table 1, which encompasses strategies to support patient resilience, harness social connections, build patient awareness and knowledge, facilitate access to support, and establish confidence and control in self-management. The strengths-based approach is in contrast to the medical model where chronic disease is traditionally focused on pathology, problems, and failures [10]. Instead, the strengths-based approach acknowledges that each individual has strengths and abilities to overcome the problems and challenges faced, and requires collaboration and cultivation of the patient’s hopes, aspirations, interests, and values. Efforts are needed to ensure that structural biases, discrimination, and disparities in the health care system also need to be identified, so all patients are given the opportunity to have a voice.

**Table 1:** Suggested strategies for “living well with CKD” using a strengths-based approach

Strengths-based approach	Suggested strategies
Build resilience	Identify or provide strategies and resources to manage stress and functioning when encountering challenges, adversity and trauma (e.g. commencement of dialysis)
Harness social connections	Facilitate connections with other patients to learn coping strategies and for support Support family members/caregivers
Build awareness and knowledge	Provide education (including practical advice) on diet and lifestyle modifications Understand, identify, and address the potential impacts of CKD (e.g. cognitive function) Encourage patients to ask questions Encourage the use of knowledge to empower and prepare for the future
Facilitate access to support	Refer to allied health care professionals (e.g. dietitian, social worker, mental health professionals, occupation therapists) Provide support that enables the patient to participate in important life activities e.g. work
Establish confidence and control in self-management	Support informed and shared decision-making (including dialysis, kidney transplantation, conservative or non-dialytic care) Encourage patients to learn to “get in tune” with what works well for them and to voice any concerns, and work together to develop better management strategies to enable patients to feel better Provide strategies to prevent or manage complications (e.g. infection) Support open communication regarding goals, concerns, and priorities

*CKD* chronic kidney disease (not receiving kidney replacement therapy)

## The role of care-partner

A care-partner is often an informal caregiver who is also a family member of the patient with CKD [11]. They may take on a wide range of responsibilities including coordinating care (including transportation to appointments), administration of treatment including medications, home dialysis assistance, and supporting dietary management. Caregivers of patients with CKD have reported depression, fatigue, isolation, and also burnout. The role of the care-partner has increasingly become more important in CKD care given the heightened complexity in communicative and therapeutic options including the expansion of telemedicine under the COVID-19 pandemic and given the goal to achieve higher life expectancy with CKD [12]. The experience of caring for a partially incapacitated family member with progressive CKD can represent a substantial burden on the care-partner and may impact family dynamics. Not infrequently, the career goals and other occupational and leisure aspects of the life of the care-partner are affected because of CKD care partnership, leading to care-partner overload and burnout. Hence, the above-mentioned principles of life participation need to equally apply to care-partners as well as all family members and friends involved in CKD care.

## Living with kidney disease in low-income regions

In low and lower-middle-income countries (LICs and LMICs) including in sub-Saharan Africa, South East Asia, and Latin America, the patient’s ability to self-manage or cope with the chronic disease varies but may often be influenced by internal factors including spirituality, belief system, and religiosity, and external factors including appropriate knowledge of the disease, poverty, family support system, and one’s grit and social relations’ network. The support system comprising healthcare providers and caregivers plays a crucial role as most patients rely on them in making decisions, and for the necessary adjustments in their health behavior [13]. In LIC regions, where there are often a relatively low number of physicians and even lower number of kidney care providers per population, especially in rural areas, a stepwise approach can involve local and national stakeholders including both non-governmental organizations and government agencies by (1) extending kidney patient education in rural areas, (2) adapting telehealth technologies if feasible to educate patients and train local community kidney care providers and (3) implementing effective retention strategies for rural kidney health providers including adapting career plans and competitive incentives.

Many patients in low resource settings present in very late stage needing to commence emergency dialysis [14]. The very few fortunate ones to receive kidney transplantation may acquire an indescribable chance to normal life again, notwithstanding the high costs of immunosuppressive medications in some countries. For some patients and care-partners in low-income regions, spirituality and religiosity may engender hope, when ill they are energized by the anticipation of restored health and spiritual wellbeing. For many patients, informing them of a diagnosis of kidney disease is a harrowing experience both for the patient (and caregivers) and the healthcare professional. Most patients present to kidney physicians (usually known as “renal physicians” in many of these countries) with trepidation and apprehension. It is rewarding therefore to see the patient’s anxiety dissipate after reassuring him or her of a diagnosis of simple kidney cysts, urinary tract infection, simple kidney stones, solitary kidneys, etc., that would not require extreme measures like kidney replacement therapy. Patients diagnosed with glomerulonephritis who have an appropriate characterization of their disease from kidney biopsies and histology, and who receive appropriate therapies and achieve remission are relieved and very grateful. Patients are glad to discontinue dialysis following resolution of AKI or acute on CKD.

Many CKD patients who have residual kidney function appreciate being maintained in a relatively healthy state with conservative measures, without dialysis. They experience renewed energy when their anemia is promptly corrected using erythropoiesis-stimulating agents. They are happy when their peripheral edema resolves with treatment. For those on maintenance hemodialysis who had woeful stories from emergency femoral cannulations, they appreciate the construction of good temporary or permanent vascular accesses. Many patients in low resource settings present in very late stage needing to commence emergency dialysis. Patients remain grateful for waking from a uremic coma or recovering from recurrent seizures when they commence dialysis.

## World Kidney Day 2021 advocacy

The World Kidney Day 2021 theme on ‘Living Well with Kidney Disease’ is deliberately chosen to have the goals to redirect more focus on plans and actions towards achieving patient-centered wellness. “Kidney Health for Everyone, Everywhere” with emphasis on patient-centered wellness should be a policy imperative that can be successfully achieved if policy makers, nephrologists, health care professionals, patients, and care partners place this within the context of comprehensive care. The requirement of patient engagement is needed. The WHO in 2016 put out an important document on patient empowerment (WHO 2016): ‘Patient engagement is increasingly recognized as an integral part of health care and a critical component of safe people-centered services. Engaged patients are better able to make informed decisions about their care options. In addition, resources may be better used if they are aligned with patients’ priorities and this is critical for the sustainability of health systems worldwide. Patient engagement may also promote mutual accountability and understanding between patients and health care providers. Informed patients are more likely to feel confident to report both positive and negative experiences and have increased concordance with mutually agreed care management plans. This not only improves health outcomes but also advances learning and improvement while reducing adverse events.’ In the ISN Community Film Event at the World Congress of Nephrology (WCN) 20 (ISN Community Film Event 2020), it is good to see a quote in the film from patients: “Tell me. I will forget; Show me. I will remember; Involve me. I will understand.” ISN Global Kidney Policy Forum 2019 included a patient speaker Nicki Scholes-Robertson from New Zealand: ‘Culturally appropriate and sensitive patient information and care are being undertaken in New Zealand to fight inequities in kidney health, especially in Maori and other disadvantaged communities.’

World Kidney Day 2021 would like to promote to the policy makers on increasing focus and resources on both drug and non-drug programmes in improving patient wellness. Examples include funding for erythropoiesis-stimulating agents and anti-pruritic agents for managing anemia and itchiness respectively, just to name but a few [15, 16]. Home dialysis therapies have been consistently found to improve patient autonomy and flexibility, quality of life in a cost-effective manner, and enhancing life participation. Promoting home dialysis therapies should tie in with appropriate ‘assisted dialysis’ programs to reduce patient and care partner fatigue and burnout. Also, examples like self-management programs, cognitive behavioral therapy, and group therapies for managing depression, anxiety, and insomnia should be promoted before resorting to medications [17]. The principle of equity recognizes that different people with different levels of disadvantage require different approaches and resources to achieve equitable health outcomes. The kidney community should push for adapted care guidelines for vulnerable and disadvantaged populations. The involvement of primary care and general physicians, especially in LICs and LMICs, would be useful in improving the affordability and access to services through the public sector in helping the symptom management of CKD patients and improving their wellness. In the overall wellness program for kidney disease patients, the need for prevention should be reiterated. Early detection with a prolonged course of wellness despite kidney disease, after an effective secondary prevention program, should be promoted [18]. Prevention of CKD progression can be attempted by lifestyle and diet modifications such as a plant-dominant low protein diet and by means of effective pharmacotherapy including administration of sodium-glucose transport protein 2 (SGLT2) inhibitors [19]. WKD 2021 continues to call for increased awareness of the importance of preventive measures throughout populations, professionals, and policy makers, applicable to both developed and developing countries [18].

## Conclusions

Effective strategies to empower patients and their care-partners strive to pursue the overarching goal of minimizing the burden of CKD-related symptoms in order to enhance patient satisfaction, health-related quality of life, and life participation. World Kidney Day 2021 theme on ‘Living Well with Kidney Disease” is deliberately chosen to have the goals to redirect more focus on plans and actions towards achieving patient-centered wellness. Notwithstanding the COVID-19 pandemic that had overshadowed many activities in 2020 and beyond, the World Kidney Day Steering Committee has declared 2021 the year of “Living well with Kidney Disease” in an effort to increase education and awareness on the important goal of effective symptom management and patient empowerment. Whereas the World Kidney Day continues to emphasize the importance of effective measures to prevent kidney disease and its progression, [18] patients with preexisting kidney disease and their care-partners should feel supported to live well through concerted efforts by kidney care communities and other stakeholders throughout the world even during a world-shattering pandemic as COVID-19 that may drain many resources [20]. Living well with kidney disease is an uncompromisable goal of all kidney foundations, patient groups, and professional societies alike, to which the International Society of Nephrology and the International Federation of Kidney Foundation World Kidney Alliance are committed at all times.

## Declarations

### Publication information

This article is being published in Kidney International and reprinted concurrently in several journals. The articles cover identical concepts and wording, but vary in minor stylistic and spelling changes, detail, and length of manuscript in keeping with each journal’s style. Any of these versions may be used in citing this article. Note that all authors contributed equally to the conception, preparation, and editing of the manuscript.
