# Correction to: Living Well with Kidney Disease by patient and care-partner empowerment: Kidney Health for Everyone Everywhere

**DOI:** 10.1007/s40620-021-01073-3

**Published:** 2021-05-25

**Authors:** Kamyar Kalantar-Zadeh, Philip Kam-Tao Li, Ekamol Tantisattamo, Latha Kumaraswami, Vassilios Liakopoulos, Siu-Fai Lui, Ifeoma Ulasi, Sharon Andreoli, Alessandro Balducci, Sophie Dupuis, Tess Harris, Anne Hradsky, Richard Knight, Sajay Kumar, Maggie Ng, Alice Poidevin, Gamal Saadi, Allison Tong, for the World Kidney Day Steering Committee

**Affiliations:** 1 The International Federation of Kidney Foundation – World Kidney Alliance (IFKF-WKA), Division of Nephrology, Hypertension and Kidney Transplantation, University of California Irvine, Orange, CA USA; 2 Department of Medicine and Therapeutics, Carol and Richard Yu PD Research Centre, Prince of Wales Hospital, Chinese University of Hong Kong, 30–32 Ngan Shing Street, Shatin, New Territories, Hong Kong, China; 3 Division of Nephrology, Hypertension and Kidney Transplantation, Department of Medicine, University of California Irvine School of Medicine, Orange, CA USA; 4 Tanker Foundation, Chennai, India; 5 Division of Nephrology and Hypertension, 1St Department of Internal Medicine, AHEPA Hospital, Aristotle University of Thessaloniki, Thessaloniki, Greece; 6 Hong Kong Kidney Foundation and the International Federation of Kidney Foundations – World Kidney Alliance, The Jockey Club School of Public Health and Primary Care, The Chinese University of Hong Kong, Hong Kong, China; 7 Renal Unit, Department of Medicine, College of Medicine, University of Nigeria, Ituku-Ozalla, Enugu, Nigeria; 8 James Whitcomb Riley Hospital for Children, Indiana University School of Medicine, Indianapolis, IN USA; 9 Italian Kidney Foundation, Rome, Italy; 10 World Kidney Day Office, Brussels, Belgium; 11 Polycystic Kidney Disease Charity, London, UK; 12 American Association of Kidney Patients, Tampa, FL USA; 13 Hong Kong Kideny Foundation, Hong Kong, China; 14 Department of Internal Medicine, Faculty of Medicine, Nephrology Unit, Cairo University, Giza, Egypt; 15 Sydney School of Public Health, The University of Sydney, Sydney, NSW Australia

## Correction to: Journal of Nephrology (2021) 34:381–388 10.1007/s40620-021-01000-6

The Living Well with Kidney Disease by patient and care-partner empowerment: Kidney Health for Everyone Everywhere, written by Kamyar Kalantar-Zadeh, Philip Kam-Tao Li, Ekamol Tantisattamo, Latha Kumaraswami, Vassilios Liakopoulos, Siu-Fai Lui, Ifeoma Ulasi, Sharon Andreoli, Alessandro Balducci, Sophie Dupuis, Tess Harris, Anne Hradsky, Richard Knight, Sajay Kumar, Maggie Ng, Alice Poidevin, Gamal Saadi and Allison Tong for the World Kidney Day Steering Committee, was originally published electronically on the publisher’s internet portal on 06 March 2021 without open access. After publication in volume 34, issue 2, pages 381–388. With the author(s)’ decision to opt for Open Choice the copyright of the article changed on 21 May 2021 to © The Author(s) and the article is forthwith distributed under a Creative Commons Attribution 4.0 International License, which permits use, sharing, adaptation, distribution and reproduction in any medium or format, as long as you give appropriate credit to the original author(s) and the source, provide a link to the Creative Commons licence, and indicate if changes were made. The images or other third party material in this article are included in the article’s Creative Commons licence, unless indicated otherwise in a credit line to the material. If material is not included in the article’s Creative Commons licence and your intended use is not permitted by statutory regulation or exceeds the permitted use, you will need to obtain permission directly from the copyright holder. To view a copy of this licence, visit http://creativecommons.org/licenses/by/4.0/.

The original article has been corrected.

